# A pragmatic cluster randomised controlled trial of air filtration to prevent symptomatic winter respiratory infections (including COVID-19) in care homes (AFRI-c) in England: Trial protocol

**DOI:** 10.1371/journal.pone.0304488

**Published:** 2024-07-23

**Authors:** Rachel C. M. Brierley, Jodi Taylor, Nicholas Turner, Sophie Rees, Joanna Thorn, Chris Metcalfe, Emily J. Henderson, Clare Clement, Tomas J. Welsh, Karen Sargent, Gemma Morgan, Derren Ready, Dominic Mellon, Liping Wen, Ruth Kipping, Alastair D. Hay

**Affiliations:** 1 Bristol Trials Centre, Bristol Medical School, University of Bristol, Bristol, United Kingdom; 2 Population Health Sciences, Bristol Medical School, University of Bristol, Bristol, United Kingdom; 3 Research Institute for the Care of Older People, Royal United Hospital, Bath, United Kingdom; 4 Patient and Public Contributor, Bristol, United Kingdom; 5 South Gloucestershire Council, Yate, Gloucestershire, United Kingdom; 6 United Kingdom Health Security Agency (South West), Bristol, United Kingdom; 7 NIHR Health Protection Research Unit in Behavioural Science and Evaluation at University of Bristol, Bristol, United Kingdom University of the West of England, Bristol, United Kingdom; 8 University of the West of England, Bristol, United Kingdom; 9 Centre of Academic Primary Care, Bristol Medical School, University of Bristol, Bristol, United Kingdom; PLOS: Public Library of Science, UNITED KINGDOM

## Abstract

**Background:**

Respiratory tract infections are readily transmitted in care homes. Airborne transmission of pathogens causing respiratory tract illness is largely unmitigated. Portable high-efficiency-particulate-air (HEPA) filtration units capture microbial particles from the air, but it is unclear whether this is sufficient to reduce infections in care home residents. The Air Filtration to prevent symptomatic winter Respiratory Infections (including COVID-19) in care homes (AFRI-c) randomized controlled trial will determine whether using HEPA filtration units reduces respiratory infection episodes in care home residents.

**Methods:**

AFRI-c is a cluster randomized controlled trial that will be delivered in residential care homes for older people in England. Ninety-one care homes will be randomised to take part for one winter period. The intervention care homes will receive HEPA filtration units for use in communal areas and private bedrooms. Normal infection control measures will continue in all care homes. Anonymised daily data on symptoms will be collected for up to 30 residents. Ten to 12 of these residents will be invited to consent to a primary care medical notes review and (in intervention homes) to having an air filter switched on in their private room. The primary outcome will be number of symptomatic winter respiratory infection episodes. Secondary outcomes include specific clinical measures of infection, number of falls / near falls, number of laboratory confirmed infections, hospitalisations, staff sickness and cost-effectiveness. A mixed methods process evaluation will assess intervention acceptability and implementation.

**Discussion:**

The results of AFRI-c will provide vital information about whether portable HEPA filtration units reduce symptomatic winter respiratory infections in older care home residents. Findings about effectiveness, fidelity, acceptability and cost-effectiveness will support stakeholders to determine the use of HEPA filtration units as part of infection control policies.

## Introduction

Respiratory tract infections (RTIs) are a leading cause of unplanned primary [1] and secondary [2] care contacts and loss of disability adjusted life years [3] worldwide. Around 10% of adults in the United Kingdom (UK) aged 65–85 years live in care homes (representing 2.5% of the UK population) [4]. Infections are common [5] and easily transmitted in care homes. Care home residents are twice as likely to receive an antibiotic prescription than their independently living peers [6], which increases the risk of antimicrobial resistance which is a major public health challenge [7–9]. Preventing RTIs in this population fits with Public Health England’s strategy of reducing use of antibiotics to prevent the development of further antimicrobial resistance [10].

Transmission of respiratory infection takes place via three principle mechanisms: (i) airborne droplets containing microbes generated during talking, coughing and sneezing; (ii) direct contact, via contaminated hands or blood/bodily fluid exchange, with staff often being unwitting vectors [11, 12]; and (iii) indirect contact via contaminated inanimate objects (e.g. door handles) [13]. There are no data regarding the relative contribution of different transmission modes, but the frequency of RTIs in care homes suggest airborne transmission is important [14]. Existing infection control methods focus on interrupting direct and indirect mechanisms, with evidence suggesting hand hygiene is effective. [15]. However, airborne transmission remains largely unmitigated beyond the use of masks as source protection and improving ventilation where possible [16]. Microbes can remain airborne for several days (up to one week) [17]. Isolating residents is logistically difficult and cannot protect other residents prior to symptom onset when many illnesses are most infectious. Isolating residents also has a detrimental impact on their physical and mental health [18, 19]. Vaccination can only reduce the transmission of specific infections, where they prevent carriage and onward spread.

Portable high efficiency particulate air (HEPA) filtration units (initially developed to remove vehicle emissions and pollen) trap particles ≥20nm. Bacteria and viruses, including influenza viruses and SARS-CoV-2, are all larger than 20nm, suggesting HEPA filtration could interrupt RTI transmission. [17] It is clear HEPA filtration units clean air (for example, one manufacturer demonstrated its product capable of removing >99% of virus particles from a room-sized test chambers in 20 minutes) [20]. However, a recent systematic review [21] found 32 studies (published after 1970) investigating whether air treatment technologies, including HEPA filtration units, in indoor settings (shared by ≥5 people for >20 hours per week). This review found no evidence that air treatment prevented the incidence of respiratory or gastrointestinal infections. However, none of the included studies assessed the impact of HEPA filtration units on respiratory infections in care home settings.

In summary, portable HEPA filtration units can capture relevant airborne particles, but there is an absence of evidence as to whether they prevent symptomatic RTIs in care home residents or other indoor environments. It is also unclear whether portable HEPA filtration units cause harm, e.g. by increasing trip hazards. To our knowledge, there is no other trial of a similar intervention in a similar setting. Research is urgently needed to help health and social care providers understand whether portable HEPA filtration units are clinically and cost-effective in reducing burden of ill health from respiratory and other infections [22].

## Materials and methods

The analysis and presentation of the trial findings will be in accordance with Consolidated Standards of Reporting Trials guidelines extension to cluster trials and guided by a pre-specified statistical analysis plan. The trial was prospectively registered (ISRCTN63437172) and the full protocol is publicly available via the funder website (https://fundingawards.nihr.ac.uk/award/NIHR129783).

### Design and setting

AFRI-c is a multi-centre, parallel group, two-arm, cluster randomised trial of portable HEPA filtration units versus no HEPA filtration units for reducing symptomatic winter respiratory infections (including COVID-19) in care home residents. The trial will be delivered over three winters (between September 2021-May 2024). Care homes will be randomised in a 1:1 ratio. The trial will include a mixed method process evaluation and economic evaluation. The design of the trial was informed by Patient and Public Involvement (PPI).

### Population

This trial will be delivered through care homes providing personal care to people predominantly over the age of 65 years in England. The trial population is older people residing in a care home. Residential care homes, with or without nursing or dementia care, will be included. We will approach care homes via the NIHR Enabling Research In Care Homes (ENRICH) clinical research network. Additional care homes will be identified via other routes, if required. The list of trial sites is available on request.

### Eligibility criteria

Care homes will be eligible to participate if they meet following criteria:

- have capacity for ≥20 residents in single bedrooms (shared bathrooms are permitted).
- predominantly focuses on care for older people (residential / nursing home)
- willing to maintain a register of all residents throughout the trial period
- willing to invite residents to receive air filtration units / accept medical notes review until the required number of residents have consented
- willing to provide anonymised resident infection data, administer brief resident and staff questionnaires, and respond to data queries
- care home owner* gives permission to take part in trial
- willing to commit to installing HEPA filtration units in care home if allocated to the intervention group
- willing to commit to not installing HEPA filtration units if allocated to control group

*where care homes are part of a large chain or charity funded, an alternative appropriate person must give permission (e.g. Chair of Trustee Board or Regional Manager).

Care homes would not be eligible to take part in the trial if they meet any of the following criteria:

- Rated on Care Quality Commission website as ‘inadequate’ or ‘requiring improvements’
- ≥10% private bedroom use of portable HEPA filtration devices
- Participating in a competing care home level study. Competing studies are those that may prevent infections or reduce infection severity or have high research burden for care home staff / residents.

There will be two levels of data collection in this trial. Anonymised daily data will be collected from up to 30 randomly selected residents. There will also be a group of approximately 10–12 consented residents in each care home. Residents will be invited to consent to provide identifiable data, access to their medical records and (if in an intervention care home) have an air filtration unit switched on in their private bedroom for the winter period.

Residents will be eligible for any data collection if they are expected to reside in the care home for at least one month of the care home data collection period. Residents will be ineligible for all data collection if they have a terminal illness with death expected within seven days.

In addition, residents will be eligible for consent if they are expected to reside in a single occupancy bedroom for ≥1 month of the data collection period and are able to give informed consent or, if lacking capacity, have a consultee willing to provide advice on their behalf. Residents will be ineligible for consent if they are participating in any other study which involves an intervention where taking part in both could adversely affect the resident or AFRI-c (to be decided by the trial team) and/or have a terminal illness (death expected within seven days).

### Objectives

The trial aims to evaluate the effectiveness and cost-effectiveness of portable HEPA filtration units on preventing symptomatic respiratory infections in care home residents. We hypothesise that using HEPA filtration units will remove enough microbes from the circulating air and exposed surfaces to reduce the spread of infection in care homes. The primary objective is to investigate the effect of using portable HEPA filtration units in resident bedrooms, communal areas and staff rooms on symptomatic winter respiratory infection episodes (including COVID-19) in care home residents.

The secondary objectives are:

1. To determine the effect of HEPA filtration units installed in resident bedrooms, communal and staff rooms during the winter months versus no HEPA filtration units **on residents****’**:
  - Respiratory infections (episodes^ii^ and symptomatic days^i, ii^)
  - Fever and/or delirium and/or acute deterioration in physical ability (episodes^i, ii^ and symptomatic days^i, ii^)
  - Gastro-intestinal infection (episodes^i, ii^ & symptomatic days^i, ii^)
  - Antibiotic use (courses prescribed^i^/days consumed^i, ii^)
  - Number of falls or near falls^i, ii^
  - Possible COVID-19 and Influenza-like illness infection episodes^i, ii^
  - Polymerase Chain Reaction (PCR) confirmed SARS-CoV-2 infections^i, ii^
  - PCR (or other test) confirmed influenza A&B infections^i, ii^
  - Other microbiologically confirmed infections including streptococcal, meningococcal, respiratory syncytial virus, norovirus and human metapneumovirus infections^i, ii^
  - General Practitioner (GP) diagnosed respiratory (including COVID-19), gastrointestinal, skin and urinary infections^i^
  - Perception of care home environment^i^
  - Hospitalisations (all cause, and potentially air filter preventable)^i^
  The indicated secondary outcomes will be collected and analysed for subsets of residents within the care homes as denoted (consented residents—i, non-consented residents—ii).
2. To determine the effect of HEPA filtration units versus no HEPA filtration units installed during winter months **on staff members****’**:
  - Sickness days off work
  - Change over time in staff confidence in, and use of, infection prevention and control strategies.
  - PCR confirmed SARS-CoV-2 infections
  - PCR (or other test) confirmed influenza A&B infections
  - Other microbiologically confirmed infections including streptococcal, meningococcal, respiratory syncytial virus, norovirus and human metapneumovirus infections
  - Perception of care home environment
3. To determine the cost-effectiveness of air filter use in reducing symptomatic respiratory infection episodes (to include hospital admissions, deaths, use of primary and community care).
4. To identify the views of care homes, local authority and Clinical Commissioning Group commissioners on intervention maintenance, sustainability and possible dissemination.
5. In the intervention arm:
  - To explore staff, resident and relatives of residents’ attitudes to and perceptions and experiences of HEPA filtration units and determine factors influencing their use, including acceptability, satisfaction and potential benefits and harms.
  - To assess adherence to the intervention.

**Fig 1:**
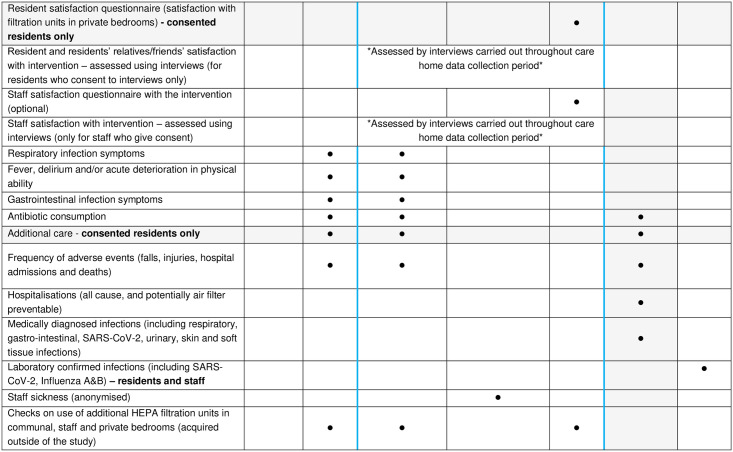
SPIRIT Schedule of Enrolment with care home and resident-related activites.

See Fig 1 for more details on timing of trial procedures and data collection.

### Randomisation, participant identification and consent

**Fig 2:**
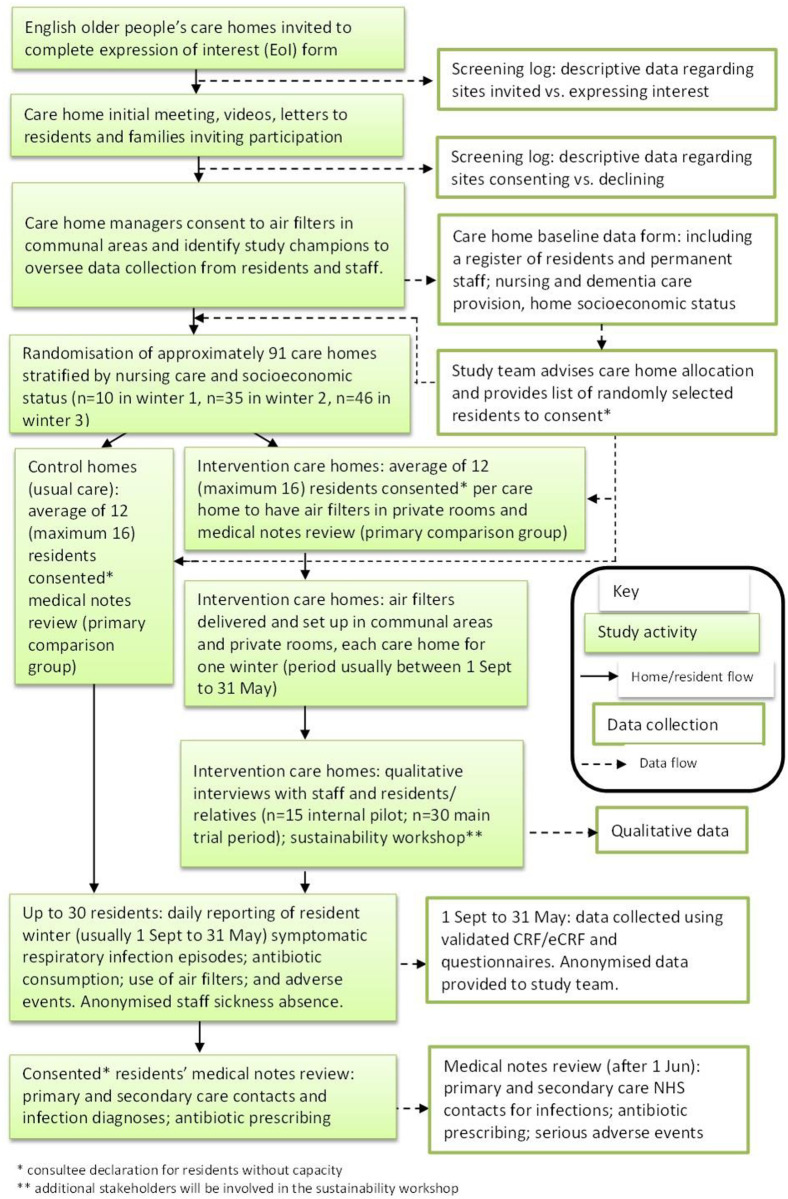
Trial flowchart.

Care homes will be screened to ensure they are eligible to participate. A meeting will be held with the care home owner /manager (or both) to ensure they understand what is involved in taking part in the trial. Once the care home has signed a contract to confirm participation in the research, they will be randomly allocated to the control or intervention group. (See Fig 2 for details.)

Before the trial starts, care homes will be asked to share information about the trial with their staff, residents and families. Care home staff will record details of anyone choosing to opt-out of participating in the research, these individuals will not be included in data collection or approached for consent. Care homes will provide and maintain a register with minimal information on each of their residents (including information about eligibility and opt-outs).

Residents will be randomly selected for participation and will be approached by care home staff to ascertain interest in the trial. In line with the 2005 Mental Capacity Act [23] capacity is assumed unless assessed otherwise. Where there is uncertainty regarding capacity to consent, formal determination of this will be undertaken. If the resident is found to lack capacity, a personal consultee (relative or close friend) will be identified by the care home staff to provide advice on whether the resident would have wanted to take part in the research. Where a personal consultee is not available, a nominated consultee (someone with a professional relationship to the resident who is independent from the trial and the care home) will be sought. The appropriate information sheet (for residents or consultees), developed with the AFRI-c PPI group, will be provided.

Informed consent (or advice from consultees for residents lacking capacity to consent for themselves) will be received by the research nurses / clinical research practitioners in the trial team, or Clinical Research Network Delivery teams, or trained care home staff. Consent (or advice from consultees) will be documented on paper or electronic consent forms.

During the enrolment process, residents (or consultees) will be asked whether they would be willing to participate in interviews for the embedded qualitative research. Separate verbal consent will be received from staff, residents and consultees participating in the qualitative interviews by the qualitative researcher before the start of each interview.

### Intervention

Both intervention and control care homes will continue with their usual infection control and cleaning processes. In addition, intervention care homes will receive HEPA filtration units for use in private and communal rooms.

We are conducting the trial using Philips^™^ HEPA filtration units: AC3033 for communal rooms and AC2936/33 for private bedrooms. The units were selected, following preparatory discussions with care homes and PPI, as they were relatively robust and unlikely to fall over easily. Each unit has a filter consisting of three layers. The outer layer captures visible particles and requires vacuuming when the brush indicator light turns on. The inner filters are self-contained, capturing particles ≥3nm, and will not ‘leak’ viruses or bacteria during vacuuming. Consideration was given to whether to use an air filtration unit without an internal filter as a sham, but it was decided that increased circulation of unfiltered air could increase the risk of infection transmission.

Enough HEPA filtration units will be provided to care homes to allow one unit per consented resident room and up to five units for the communal areas in the care home. Floor plans and communal room dimensions (to enable calculation of approximate room volume) will be submitted by the care home. Measurements will not be required for private bedrooms as they are less variable in size and layout than communal areas.

The placement of the units will be discussed with each intervention care home team following receipt of dimensions and floor plans. Sufficient units will be placed in each communal area of the care home to enable a clean air delivery rate of ≥160m^3^/hr. Where there are more communal areas than the five HEPA filtration units can cover, units will be placed in the most frequently used communal areas in the care home wherever possible. Units are not placed in kitchens as these are not usually frequently used by residents.

Care homes will be provided with guidance about where to place the units to maximise efficiency of air filtration, based on manufacturer guidance (e.g. ≥20 cm away from walls / objects and away from externally opening doors and windows where possible). They will also be advised to place the units to minimise the risk of trip hazards from the units. When data collection starts, care homes will be required to enter at least two days of data collection before they switch on the filtration units. They will be advised to keep all units always switched on after this point. The care homes will be asked to keep the units on a single mode continuously (sleep mode in bedrooms and mode one in communal rooms). Local research delivery teams will be asked to support care home staff with installation of the units.

Care homes allocated to receive the intervention will be asked to carry out daily checks on each HEPA filtration unit to ensure that they are still switched on, in the correct location and in the correct mode. They will be asked to complete further details if any of the details are not as expected. Adherence monitoring will also be undertaken by providing an energy meter with each unit to monitor electricity usage for costing and compliance monitoring for the trial. Care home staff will be asked to submit weekly energy meter readings, which will show cumulative energy use data over time. Labels will be provided to remind care home staff and residents that HEPA filtration units must not be switched off.

### Criteria for discontinuing or modifying allocated interventions

Participants have the option of stopping participation in any of the following parts of the research: daily data collection by care home staff, having a HEPA filtration unit in their private room (for intervention care home residents), primary care notes review and linkage to UK Health Security Agency (UKHSA) data. If participants stop participating in any part of the research or leave the trial due to death or moving permanently away from the care home, this will be documented and we will seek to consent another resident to replace them. Where a resident loses capacity and a suitable consultee cannot be found, that resident will stop participation in the trial.

Where residents stop participation (for any reason) in an intervention care home, the filtration units will be switched off temporarily whilst a randomly selected replacement participant is consented. The internal filter will be replaced, and local infection control measures followed before the filtration unit is switched on in the new participant’s private bedroom to minimise risk of cross contamination. There must be two days of baseline data collection before the unit is switched on in the replacement participant’s private room.

Where care home staff consider the intervention as unsuitable for any residents (for example, if they become disorientated and damage the unit), there will be a discussion with the trial team and the consultee (if appropriate). If necessary, the participant may stop participating in the research.

Care homes have the right to withdraw from the trial if they wish.

### Outcome measures

#### Primary outcome

The primary outcome is the number of symptomatic winter respiratory infection episodes measured using electronic case report forms (CRF) completed daily by staff and/or the resident throughout the winter period. We have defined ‘winter’ as the period starting on 1 September and ending on 31 May. There are significantly higher rates of RTIs circulating in this period. We expect to detect most respiratory infection episodes in this winter period.

The definition of a symptomatic respiratory infection is “one or more new (or worsening of pre-existing) objective respiratory symptoms from the following: runny/blocked nose; sneezing; runny ear; red/sticky eye/s (part of the respiratory tract due to the lacrimal duct); hoarse voice; cough; wheeze; noisy breathing; or sputum (phlegm)”. Shortness of breath will also be collected (as it contributes to UKHSA’s definition of possible influenza-like illness) but when present in isolation will not be regarded as a symptomatic respiratory infection episode. The subjective respiratory symptoms (sore/tickly throat, earache and change in taste/smell) will similarly be collected where the resident is able to communicate their presence for sensitivity analyses.

The start and end of a discrete symptomatic respiratory infection episode will be defined as per previous studies [24–26]. The start will be the onset of two new (or worsening of pre-existing) respiratory symptoms for ≥1 day or one respiratory symptom for ≥2 days; the end will be the last symptomatic day preceding two asymptomatic days.

### Secondary outcomes

Clinical outcomes for residents have been chosen to reflect the potential effects of the HEPA filtration units and include:

- Number of days with respiratory infection symptoms (collected daily)
- Presence of fever and/or delirium and/or acute deterioration in physical ability (collected daily)
- Number of gastro-intestinal infection episodes and number of symptomatic days of gastro-intestinal infection symptoms (collected daily)
- Number of days antibiotics are consumed and prescribed (number and name -from primary care notes review)
- Number of falls/near falls (collected daily)
- Number of possible** SARS-CoV-2 infection episodes and possible** influenza-like illness episodes (collected daily)
- Number of diagnosed respiratory (including COVID-19), gastrointestinal, skin and urinary infections (from primary care notes review)
- Hospitalisations (all cause, and potentially air filter preventable—from primary care notes review)

We will link to UKHSA data (for residents and staff) after the winter period to identify the number of:

- Laboratory confirmed cases of SARS-CoV-2 and influenza A and B infections
- Other microbiologically confirmed infections as investigated by Public Health England during care home outbreaks, including streptococcal, meningococcal, respiratory syncytial virus, norovirus and human metapneumovirus infections

A cost-effectiveness analysis will be completed for the consented residents. Resident and staff questionnaires will provide information about their perception of the care home environment. Qualitative interviews with staff, residents and consultees will enable us to explore staff, resident and residents’ relatives/ friends’ attitudes to and perceptions and experiences of air filtration units and determine factors influencing their use including acceptability, satisfaction and potential benefits and harms. Fidelity to the intervention will be assessed using qualitative interviews and daily questions about the HEPA filtration units.

We will collect information about number of staff sickness days away from work due to respiratory infection. Other secondary outcomes will enable us to determine the acceptability of the intervention to all stakeholders and the potential for implementing air filtration units if the trial demonstrates they reduce infection rates.

Safety data will be collected wherever there is a serious adverse event affecting any person in the care home (resident, staff or visitor) that may be related to the research. The trial team will assess each event and submit onward reporting as required.

### Data collection and management

#### Quantitative data

Trial specific questionnaires will be used to determine care home demographics and perceptions of the care home environment and effectiveness of air filtration units (for consented residents and any staff not involved in consenting residents). All questionnaires will be completed at baseline and again at the end of the data collection period. Where possible, staff questionnaires will be completed before data collection starts. Staff questionnaire completion will be optional.

Data on resident infection symptoms, falls and near falls will be collected daily by care home staff and entered directly into an electronic CRF. Information about confirmed infections will be collected from linkage with UKHSA data. Information from the UKHSA will be linked to daily infection data for the consented residents; we will also collect anonymised datasets from the UKHSA about total confirmed infections in all residents and staff in the care home. More detailed information about infections and antibiotics will be collected for consented residents as part of the primary care medical notes review, along with information about National Health Service (NHS) resource use and hospitalisations. Data will also be collected for residents treated using the “hospital at home” service. The primary care notes review will inform the cost analysis.

Care home staff will be asked to document anonymous information about staff absences at regular intervals. Care home staff at intervention care homes will also enter information about compliance with the intervention (daily and weekly) on an electronic CRF. Data quality and completeness will be checked regularly by the central team. Where necessary queries will be issued to sites and followed through until they are resolved. Data collection forms are available on request from the trial team.

### Qualitative data

Purposive sampling will be used to select interviewees, aiming for maximum variation in terms of age, gender and ethnicity of the resident and staff role and care home size and status (public/private) for staff interviewees. A small number of interviews with staff in control care homes will provide additional information about trial processes and potential contamination. Up to 45 care home staff, residents, and relatives will be interviewed. The final sample size will be determined based on ’information power’ [27].

Interviews with residents will take place in person. Staff and consultees will be interviewed in person or remotely (telephone or video call) depending on personal preference and convenience. All interviews will be audio-recorded, transcribed verbatim and pseudonymised. All potential participants will be provided with an information sheet before being invited to give consent to take part in the interview. Care home staff will support residents by sharing participant information with them prior to the researcher visiting the site.

Wherever possible, all data (except the qualitative data) will be entered directly onto the password protected database that will only be accessible to members of the research team, held on a secure system held at the University of Bristol. Qualitative interviews will be recorded using an encrypted device and deleted once transferred to the secure University of Bristol server. Audio-recordings of interviews will be transcribed in full by a University of Bristol member of staff or a University of Bristol approved transcription service and will be pseudonymised during transcription.

Audio-recordings and transcripts will be labelled with a trial identification number and stored securely in a folder that only the qualitative research team will be able to access. With consent, anonymised quotations may be used for training, teaching, research and publication purposes for this and future studies.

### Sample size

Previous research suggests ~30% of residents will wish to take part and ~40% will leave the care home or die during the trial, [28] reflecting the frailty and vulnerability of the trial population. To maintain trial power, we will continually recruit new residents, resulting in an expected overall trial data attrition of ~20%. We consider the advantages in power outweighs the potential disadvantage arising from the unblinded recruitment of a proportion of trial participants.

Considering the available literature [12, 28–32] and based on PPI and investigator experience, we expect care home residents to experience two respiratory infections during the winter months (usually 1 September to 31 May = 273 days). Our PPI group advised that a reduction of one infection would be important to residents, relatives and staff.

Based on 90% power and an alpha of 0.05, to detect a reduction in winter respiratory infections of 1 per 242 person days, from 2 to 1 per 242 person days, assuming a coefficient of variation of 0.78 to allow for variation between care homes in infection rates, calculated from data provided by the PRINCESS study team [28], a total of 74 care homes will be required. Assuming a mean cluster size of 10 participants per care home for winters 1 & 2 and 12 for winter 3,and accounting for an attrition level as observed in PRINCESS [28], with the continual recruitment strategy as described above, each cluster will provide a mean of 1920 person days of follow up. After reviewing accumulating data with the Data Monitoring Committee (DMC) and Trial Steering Committee (TSC) on the number of follow up days, the number of care homes taking part in Winter 3 will increase from 32 to 46 to achieve the number of person days required in the original calculation. In total 91 care homes will take part in the trial.

### Allocation concealment

Randomisation will be stratified by whether care homes provide nursing care (yes/no) and socioeconomic tertile (high/medium/low) using a randomisation list pre-generated by a statistician with no other involvement in recruitment activities. The allocation sequence will be generated in permuted blocks of size two and four. The trial management team will email the stratification details for each care home to the statistician to request group allocation.

### Blinding

The lead statistician will be blinded throughout the trial. The trial statistician will perform all disaggregated analyses according to a pre-specified Statistical Analysis Plan (SAP). Two health economists based at the University of Bristol will be blinded when cleaning data and preparing the Health Economic Analysis Plan (HEAP) but will be unblinded when conducting the analysis.

### Statistical methods

#### Quantitative analysis

The primary analysis will be conducted on the consented residents under the intention to treat (ITT) principle using a mixed effects Poisson regression of number of respiratory infection episodes on allocation. We will adjust for care home nursing care provision, deprivation tertile (stratification variables), and which winter period the data was collected and will accommodate variable durations of follow-up across participants and a random effect for clustering by care home. Results will be presented as an incidence rate ratio with associated 95% confidence interval and p-value. The effect of the intervention on secondary outcomes will be investigated using the same analytical approach as the primary analysis; using mixed effects regression models that are appropriate to the nature of the outcome being analysed (e.g., a logistic model for a binary outcome and a Poisson model for count data).

A limited number of pre-specified exploratory subgroup analyses will be performed. Potential treatment effect moderators will be investigated at the cluster- and individual-levels. The impact of intervention adherence will be explored using sensitivity analysis. Sensitivity analyses may also be used to explore the potential impact of missing outcome data, with the approach taken depending on the assumptions about missingness.

### Health economic analysis

A within-trial cost-consequence analysis will be conducted, utilising primary and secondary outcome data, from the NHS and Personal Social Service and care home provider perspectives. A complementary cost-effectiveness analysis will take an NHS/ Personal Social Service perspective with the incremental cost-effectiveness ratio expressed as the cost per respiratory infection episode averted.

Healthcare resources relating to respiratory infection will include GP and community contacts, emergency visits, hospital admissions and antibiotic prescribing which will be valued using national sources of unit costs [33, 34] and/or market (actual) prices where applicable. Productivity losses relating to care home worker absence will also be considered. Intervention costs will be based on the acquisition, installation, maintenance (e.g. annual safety testing), consumables (e.g. filter replacement), air filter running costs, and depreciation of the units.

The analysis will be based on the ITT population (consented residents only) and will be guided by a pre-specified HEAP [35]. Uncertainty will be represented by cost-effectiveness acceptability curves and best practice will be followed to deal with skew, baseline imbalance, correlated costs and effects and missingness [36].

### Qualitative analysis

In the pilot phase, a rapid analysis using a framework approach [37] will be conducted to identify any implementation issues of the intervention. Findings will be fed back rapidly to the wider team, with the aim of implementing any potential improvements that are identified. Two researchers will independently listen to the recordings and enter notes or direct quotations into a matrix for rapid analysis. Fields will be pre-determined initially but will be flexible as the analysis progresses. Subsequently, reflexive thematic analysis [38, 39] will be applied to all pseudonymised transcripts. Two researchers will independently code a subset of the transcripts for initial codes and categories. These codes will then be applied to the remaining transcripts by one researcher with ongoing refinement and theme development as the analysis progresses. Normalisation process theory [25] constructs will then be used to aid further interpretation of the evolving themes.

Qualitative and quantitative data specified in the Process Evaluation Analysis Plan (PEAP) will be brought together using a triangulation protocol approach [40–42]. The SAP and HEAP will be published before analysis of the data begins. The PEAP will also be published before statistical analysis and triangulation commences.

### Trial management and oversight

The trial is managed by the Bristol Trials Centre and sponsored by University of Bristol (Red-Office@bris.ac.uk). The Sponsor does not have any involvement in the design of the trial, data collection, analysis or interpretation of the data, decision to publish or preparation of the manuscript. The Trial Management Group (TMG) is led by the Chief Investigator and comprises all investigators, the PPI representatives, the trial manager(s), the trial research nurse, and administrative staff. The TMG will be responsible for trial design, conduct, management, strategy, costs, data analyses and publication. With the support of all staff, the trial manager will operationalise TMG strategy and oversee day to day management. The TMG will meet monthly to review progress against project specific milestones.

The TSC will be chaired by an experienced public health/ geriatrician academic and at least three other independent members to include a clinical trialist, a statistician and PPI representation. Representation will be invited from the Host, Sponsor and the NIHR. An experienced care home trial statistician will chair the independent DMC, which will include at least two other independent members. The TSC and DMC will meet once prior to recruitment of the first participant and then at agreed intervals (at least annually).

### Ethical considerations and dissemination

Ethical approval for this trial was given by London—Harrow NHS Research Ethics Committee (Ref 21/HRA/4318) on 24 November 2021. Written information consent to participate will be obtained from all participants (or appropriate consultees where relevant). The central trial team will prepare amendments in discussion with the TMG. Amendments that affect sites will be communicated to the local principal investigators and site teams with instructions for implementation.

The Chief Investigator and TMG will develop a publication policy setting out the roles and responsibilities of authors preparing scientific reports of the trial findings, including a full report for the NIHR. We aim to publish a primary manuscript in a public health or medical journal, published as open access, with additional analyses described in specialty journals. Primary findings will also be presented at key meetings and conferences. We aim to disseminate the findings widely to the care home community, health care providers, policymakers and the public through a wide range of communication methods. Participant details will be anonymised in any publications that result from the trial. All data shared will be anonymised and only available (via controlled access) to other researchers who secure the necessary approvals for purposes not related to this trial.

### Trial status

Current protocol is v11.0, 18 September 2023. The first participant was recruited on 02/02/2022. Recruitment will be completed on 30 April 2024.

## Discussion

The AFRI-c trial is the first randomised controlled trial to investigate whether portable HEPA filtration units reduce symptomatic respiratory infections in residents of care homes. The trial started recruiting in February 2022 and recruitment will continue until the end of April 2024. Due to delays in opening sites in the first two winters, the cluster size was increased from ten to 12 in the third winter (2023/2024) to ensure there were sufficient data collection days to meet the requirements for the sample size calculation. The eligibility criteria were adjusted at the end of the first (pilot) winter (2021/2022) to include smaller care homes (initially care homes were only eligible if they had ≥30 residents in single rooms). This change was made on the advice of the Enabling Research In Care Homes network as smaller care homes tend to be more active in research participation. Following the pilot, we also capped the anonymised daily data collection for non-consented residents so that care homes were collecting daily data for no more than 30 residents (in the pilot this was for all residents in the care home). This change was made to reduce the burden on care homes participating in the trial.

The AFRI-c trial is designed and conducted as a pragmatic trial. This is a large cluster randomised controlled trial with broad inclusion criteria. Efforts have been made by researchers to ensure that residents without capacity to consent to their involvement are afforded the opportunity to participate. In communal rooms the intervention is assessed using clean air delivery rates, which means that different air filtration units can be used with reference to the room measurements and product specification. If the intervention is shown to be effective, we anticipate that it will be relatively straightforward to replicate in usual residential home settings in the UK and internationally.

A limitation of the trial design is that the consent process takes place after the care homes are informed of the randomised allocation. We recognise that this could affect some participants’ decisions to take part, we will therefore evaluate the difference in enrolment rates between groups. Given the trial population and the design of ongoing consent to replace any participants leaving the trial, it would not have been possible to conduct the trial without participants knowing the care home group allocation. The other main limitation is that the data entry is completed by care home staff, most of whom are research naïve. This has been managed with training and making the data collection as simple as possible. Feedback from care homes suggests that the daily data collection (for infection symptoms) is similar to data routinely collected by care home staff.

If the intervention is found to be safe, effective, and cost-effective this will have important public health policy and practice implications for infection prevention in care homes. During the trial we have received information that air filtration is already embedded in some care home infection control processes, despite the lack of evidence to date that air filtration units are an effective method of infection control. As part of the trial, we are engaging with stakeholders in discussions about who would pay, should the results show that air filtration units are effective and cost-effective. We have also embedded qualitative research on the acceptability of air filter installation to staff, residents and families in care homes.

In summary, there is an important evidence gap to address whether using HEPA filtration units in care homes could reduce respiratory infections in winter. There is pressure on care homes (including from the media and manufacturers) to invest in devices for infection control processes that may not be effective. The AFRI-c trial will provide evidence on whether using portable HEPA filtration units is effective, acceptable, feasible and cost-effective in reducing respiratory infections in older care home residents.

## Supporting information

S1 Checklist SPIRIT 2013 checklist: Recommended items to address in a clinical trial protocol and related documents*. (DOCX)

S1 File (DOCX)

S2 File (DOCX)

S3 File (DOCX)

S1 Protocol (DOCX)

## Abbreviations

CRF: Case Report Forms
DMC: Data Monitoring Committee
ENRICH: Enabling Research In Care Homes
GP: General Practitioner
HEAP: Health Economics Analysis Plan
HEPA: High Efficiency Particulate Air
ITT: Intention To Treat
NHS: National Health Service
NIHR: National Institute for Health and Social Care
PCR: Polymerase Chain Reaction
PEAP: Process Evaluation Analysis Plan
PPI: Patient and Public Involvement
RTI: Respiratory tract infections
SAP: Statistical Analysis Plan
TMG: Trial Management Group
TSC: Trial Steering Committee
UK: United Kingdom
UKHSA: UK Health Security Agency
