# Supplementary material for: A pragmatic cluster randomised controlled trial of air filtration to prevent symptomatic winter respiratory infections (including COVID-19) in care homes (AFRI-c) in England: Trial protocol
Source: PLoS One. 2024 Jul 23;19(7):e0304488. doi: 10.1371/journal.pone.0304488 (PMC11265654; doi:10.1371/journal.pone.0304488)
Supplement: S1 File — (DOCX) [file pone.0304488.s002.docx]

AFRI-c Interview Topic Guides

# Staff

**Background on participant**

- Job role and what’s involved, length of time working in care home (this and others)
- Any previous experience of research, RCTs?
- Role in AFRI-C – what activities involved in? How differs from usual role (increased workload/responsibility)?

**Views and experiences of trial and processes**

- Understanding of the AFRI-C study
- What was your first impression of the AFRI-C study?
- What do you think is the aim of the AFRI-C study?
- Why do you think we are doing this study?
- Do you see a need for the AFRI-C study? *Why/why not?* Does it fit in with existing care home priorities? - If manager – why did care home take part in the study?

*If study champion (collecting data/recruiting/consenting residents)*

- How is data collection going? Have you been able to collect the data needed? Any issues, barriers to collecting the data?
- Do you think staff movement or changes has had any impact on air filter implementation or use?

**Views and experiences of air filters**

- When selecting residents for registration, were there any factors you took into account to make a decision about each resident? If yes, what factors? Examples?
- Can you tell me what you knew about air filters before the study?
  - Where did you get information on air filters? E.g. online, family/friends/colleagues
- How would you explain the air filters and their purpose in the care home?
- What have been your first impressions of the air filters?
  - What were your expectations of them?
  - Did you have any concerns *e.g. finding a place to fit them, risk of falls*
- What has been the response from other staff, care home management, residents and family etc.? *What are their thoughts on the air filters?*
- How have the air-filters been used in the care home?
  - Where are the air-filters placed? Any issues? How many residents have them in their private rooms? Frequency of use – how often are they turned on? Why are they not turned on? Have you had any problems placing the air filters? Have they been turned off by accident and why? Falls/trips?
- Have any other residents put air filters in their rooms?
- What effect do you think the air filters have on residents? (Can they still get respiratory tract infections if they have an air filter?
- How does having the air filters in the care home affect your reporting of symptoms? Do you still report symptoms if a resident has an air filter?
- How has having air filters in the care home affected how staff think about infection prevention and control? do they think they don’t need to worry, or worry more?
  - What infection control measures are you using this winter? Are there any restrictions e.g. to visiting? Perceived effect on respiratory infections?
  - When I asked residents about their concerns about infections, most of them said they were not particularly worried about it anyway – what’s your view on that?
- Do you think having air filters in the home changes how you feel about residents coming back from a hospital stay? Why?
- What has been the impact of the air filters on staff roles and time?
- Can you tell me how/if things have changed over the last few years as we get further away from the start of the covid pandemic in terms of infection control measures.
  - What is the situation now compared to last winter? (PPE, measures, experiences, fears)

**Impact of air filters (including work required to implement the air filters)**

- What has been involved for you and your home to set up and use the air filters? How have staff worked with each other and residents? *(prompt install, turn on and keep on/turn off, change filters).* Explore impact on working practices, time.
- How well prepared do you think the home was for using the air filters? Enough information and support?
- What would you say are the benefits of the air filters?
- Do you think there are any harms caused by the air filters?
- What has worked well or has been successful when using the air filters?
- What hasn’t worked well? Have there been any difficulties when using the air filters (why & how managed this)? Probe around:
  - Remembering to use it?
  - Practical issues?
- Do you have any suggested changes to how the air filters are used in care homes? How can implementing and using them be improved?

**Views of potential for change in practice**

- Any barriers/challenges to further/wider use?
- Can you think of any changes that would be needed for air filters to be used more widely in care homes? (any change, if yes, describe)
- Practical issues – care home level?
- Has the care home or anyone in the home bought air filters?
- Given you felt certain residents were not suitable for having air filters, how do you think that will affect implementation? What could be done to make air filters more suitable for those types of residents?

**Any other issues**

- Any other issues the participant would like to raise?

# Personal consultees

**Participant Background**

Can you tell me a little bit about yourself – relationship to resident, how often see, speaks to resident – visits the resident?

**Views and understanding of study**

- Can you tell me about when you first heard about the AFRI-C study? Where, when, who?
  - How was the study explained to you?
- What did you think about the study? good idea/bad/ any concerns?
- Are infections something you worry about in relation to your relative being in a care home?
- Do you think COVID changed that?
- How has your perspective shifted since the start of covid – do you think it’s back to how it was regarding infection control measures?
- What is your understanding of the AFRI-C study? What are the researchers trying to do and why?
- Did the study make sense to you? probe why, why not – anything worried about?
- Could you tell me your thoughts on being involved in the study? Anything worried about? Anything they like about it?
- Did you talk to [resident] about it?
- Why did they decide to become a personal consultee?
- What’s involved?
- Thoughts on the consent process? Explore levels of capacity for consent and their role

**Views and experiences of the air filters**

- Where in the room is the air filter – can you please describe it to me as obviously I can’t go and see for myself
- Thoughts on air-filters – tell me about what you think they are and what they are for?
- Are they aware of the air filter in the room? Who set up the filter? How long been in room? Do you know if it’s been switched on? Who turns it on/off? Does the resident need to do anything? if yes, what, is this ok? Any issues?
- What do they think the benefits of the air filters are? Do they think the air filters can reduce infections?
- What do they like about the air filter? Explore
- What don’t they like about the air filter? Explore
- Has/can the air filters cause any harm?
- What do others think about the air filters? other residents, family, friends – staff? Have they talked about them with the resident? Response from resident?
- Are they happy to continue to have the air filter in the resident’s room? for resident to continue to be part of the study? Why?
- Is there anything they would change about having the air-filters in the resident’s room?
- Would they recommend the air filters to family and friends?
- Do you think it’s having an impact?
- Do you have any thoughts about, if we find them to be effective, how they should be rolled out?
  - Who should pay?
  - Anything you would change about them in the care setting?

**Any other issues**

- Any other issues the participant would like to raise/discuss further?

# Residents

**Views and understanding of the study**

| **Main question** | **Optional if able** |
| --- | --- |
| Can you tell me why this machine [gesture to it] is in the room?  Prompt: What do you think it does? |  |
| [Explain study if needed: We are putting air filters in some care homes and in some rooms to see if they reduce the number of infections (like colds, flu, covid).]  Do you think it’s a good idea to see if using air filters in care homes results in fewer infections? | Do you worry about getting a cold or chest infection or covid?  Follow-ups: Has covid changed your perspective on that? Do you take any precautions about these kinds of infections? |
| What did you think about the study when you were told about it? | Prompt – did you think it was a good/bad idea?  What did you think of the documents you were given? |
|  | Was there anything you were worried about in relation to the study? |
|  | Why did you decide to take part? |
|  | Did you talk to anyone else about the study before you decided to join?  Prompts: staff/resident [use names], family members, friends?  [If yes, try to ascertain who and whether can also interview them if sounds relevant] |
| Does someone [use name if known] ask you about cold symptoms every day? | If yes: Is that okay, is it bothersome or easy to answer? |
|  | Is there anything about being in the study you don’t like or are worried about? |

**Views and experiences of the air filters**

| **Main question** | **Optional if able** |
| --- | --- |
|  | How did you feel when you found out you were going to have an air filter in your room?  Prompts: Was there anything that worried you? |
| What do you think about the air filter now it’s in your room? |  |
| What do you think might be the benefits of the air filters?  And what might be the potential harms? |  |
| Is there anything you like about the air filter? |  |
| Is there anything you don’t like about the air filter? |  |
| Are you happy to keep the air filter in your room? |  |
| Is there anything you would change about having the air filter in your room?  Prompts: positioning, noise, cold |  |
| How easy was it to find a place to put the air filter in your room? | Have you moved the air filter since it was put in?  Did you have to make any changes in your room? |
|  | Does someone come in and check the air filter? |
|  | Have you seen the air filter in the [name communal area]? What are your thoughts about air filters in the communal areas?  Prompt: is there anything you would change about those? |
|  | Have you spoken to [e.g. resident names]/family/friends about the filter in your room? What do they say about it? |

**Any other issues**

- Is there anything else about the air filters or the research you’d like to raise/discuss further?
