# Supplementary material for: A pragmatic cluster randomised controlled trial of air filtration to prevent symptomatic winter respiratory infections (including COVID-19) in care homes (AFRI-c) in England: Trial protocol
Source: PLoS One. 2024 Jul 23;19(7):e0304488. doi: 10.1371/journal.pone.0304488 (PMC11265654; doi:10.1371/journal.pone.0304488)
Supplement: S2 File — (DOCX) [file pone.0304488.s003.docx]

**A study to see if Air Filtration prevents winter coughs, colds, flu and COVID-19 in care homes (AFRI-c)**

**RESIDENT QUESTIONNAIRE CONTENT**

The following questionnaire content will be displayed on the AFRI-c study database for entry by the care home staff. The questions will be asked by the care home staff to the resident/consultee and entered into the database by the care home staff member.

**BASELINE QUESTIONNAIRE**

| Question | Answer |
| --- | --- |
| I believe infections can be spread through the air, for example as droplets from a sneeze or cough | 1, Strongly disagree 2, Slightly disagree 3, Not sure 4, Slightly agree 5, Strongly agree 6, Not recorded/missing |
| I believe air filters reduce infections being spread through the air | 1, Strongly disagree 2, Slightly disagree 3, Not sure 4, Slightly agree 5, Strongly agree 6, Not recorded/missing |
| How satisfied have you been with the quality of your sleep? | 1, Very satisfied 2, Satisfied 3, Not sure 4, Dissatisfied 5, Very dissatisfied 6, Not recorded/missing |
| How would you describe your satisfaction with your care home in terms of…. |  |
| Care home temperature | 1, Very satisfied 2, Satisfied 3, Not sure 4, Dissatisfied 5, Very dissatisfied 6, Not recorded/missing |
| Odours | 1, Very satisfied 2, Satisfied 3, Not sure 4, Dissatisfied 5, Very dissatisfied 6, Not recorded/missing |
| Air quality | 1, Very satisfied 2, Satisfied 3, Not sure 4, Dissatisfied 5, Very dissatisfied 6, Not recorded/missing |
| Other |  |
| Is there anything else you would like to tell us about the things in this questionnaire? | Free text |
| How many days per week would you expect to a visit communal room ? | 1, Integer  2, Don't know |

**FOLLOW UP QUESTIONNAIRE – MARCH**

| Question | Answer |
| --- | --- |
| I believe infections can be spread through the air, for example as droplets from a sneeze or cough | 1, Strongly disagree 2, Slightly disagree 3, Not sure 4, Slightly agree 5, Strongly agree 6, Not recorded/missing |
| I believe air filters reduce infections being spread through the air | 1, Strongly disagree 2, Slightly disagree 3, Not sure 4, Slightly agree 5, Strongly agree 6, Not recorded/missing |
| How satisfied have you been with the quality of your sleep? | 1, Very satisfied 2, Satisfied 3, Not sure 4, Dissatisfied 5, Very dissatisfied 6, Not recorded/missing |
| How would you describe your satisfaction with your care home in terms of…. |  |
| Care home temperature | 1, Very satisfied 2, Satisfied 3, Not sure 4, Dissatisfied 5, Very dissatisfied 6, Not recorded/missing |
| Odours | 1, Very satisfied 2, Satisfied 3, Not sure 4, Dissatisfied 5, Very dissatisfied 6, Not recorded/missing |
| Air quality | 1, Very satisfied 2, Satisfied 3, Not sure 4, Dissatisfied 5, Very dissatisfied 6, Not recorded/missing |
| Would you like to tell us anything else about the above items? | 1, Yes- Free text bow, 2, No |
| The following questions are for residents in intervention care homes ONLY | |
| Since the air filter was install in your private room: | No input |
| How would you describe your overall satisfaction with the air filter in your room? | 1, Very satisfied 2, Satisfied 3, Not sure 4, Dissatisfied 5, Very dissatisfied 6, Not recorded/missing |
| Do you have a preferred mode for your air filter to be on? | 1, Yes  2, No 6, Not recorded/missing |
| If YES, what mode do you prefer during the daytime? | Drop Down |
| If YES, what mode do you prefer during the night-time? | Drop Down |
| Would you like to tell us anything else about the filters | 1, Yes- Free text bow, 2, No |
