# Supplementary material for: A pragmatic cluster randomised controlled trial of air filtration to prevent symptomatic winter respiratory infections (including COVID-19) in care homes (AFRI-c) in England: Trial protocol
Source: PLoS One. 2024 Jul 23;19(7):e0304488. doi: 10.1371/journal.pone.0304488 (PMC11265654; doi:10.1371/journal.pone.0304488)
Supplement: S3 File — (DOCX) [file pone.0304488.s004.docx]

**A study to see if Air Filtration prevents winter coughs, colds, flu and COVID-19 in care homes (AFRI-c)**

**STAFF QUESTIONNAIRE CONTENT**

This questionnaire asks about your experiences of infection prevention and control and your care home environment. Your answers will help us to understand the views of care home staff. We will ask you to complete this questionnaire twice, once at the beginning of winter (usually in September) and again in March. Completing this questionnaire is optional and your care home will not know whether or not you have completed it.

The following questionnaire content will be displayed on the AFRI-c study database. The questionnaire will be accessed by staff via a link to an online data collection form.

**BASELINE QUESTIONNAIRE**

| Question | Answer |
| --- | --- |
| I know how to prevent infection transmission | 1, Strongly disagree 2, Slightly disagree 3, Not sure 4, Slightly agree 5, Strongly agree 6, Not recorded/missing |
| Lack of time or facilities sometimes prevents me from following infection control procedures | 1, Strongly disagree 2, Slightly disagree 3, Not sure 4, Slightly agree 5, Strongly agree 6, Not recorded/missing |
| I am confident my use of infection control procedures is effective | 1, Strongly disagree 2, Slightly disagree 3, Not sure 4, Slightly agree 5, Strongly agree 6, Not recorded/missing |
| I believe infections can be spread through the air, for example as droplets from a sneeze or cough | 1, Strongly disagree 2, Slightly disagree 3, Not sure 4, Slightly agree 5, Strongly agree 6, Not recorded/missing |
| I believe air filters reduce infections being spread through the air | 1, Strongly disagree 2, Slightly disagree 3, Not sure 4, Slightly agree 5, Strongly agree 6, Not recorded/missing |
| How would you describe your satisfaction with your care home in terms of…. |  |
| Care home temperature | 1, Very satisfied 2, Satisfied 3, Not sure 4, Dissatisfied 5, Very dissatisfied 6, Not recorded/missing |
| Odours | 1, Very satisfied 2, Satisfied 3, Not sure 4, Dissatisfied 5, Very dissatisfied |
| Air quality | 1, Very satisfied 2, Satisfied 3, Not sure 4, Dissatisfied 5, Very dissatisfied 6, Not recorded/missing |
| Other Comments |  |
| Is there anything else you would like to tell us about relating to this questionnaire? | Free text |
| Name of care home | Free text |
| Date completed | dd/mm/yyyy |

**FOLLOW UP QUESTIONNAIRE – MARCH**

| Question | Answer |
| --- | --- |
| I know how to prevent infection transmission | 1, Strongly disagree 2, Slightly disagree 3, Not sure 4, Slightly agree 5, Strongly agree 6, Not recorded/missing |
| Lack of time or facilities sometimes prevents me from following infection control procedures | 1, Strongly disagree 2, Slightly disagree 3, Not sure 4, Slightly agree 5, Strongly agree 6, Not recorded/missing |
| I am confident my use of infection control procedures is effective | 1, Strongly disagree 2, Slightly disagree 3, Not sure 4, Slightly agree 5, Strongly agree 6, Not recorded/missing |
| I believe infections can be spread through the air, for example as droplets from a sneeze or cough | 1, Strongly disagree 2, Slightly disagree 3, Not sure 4, Slightly agree 5, Strongly agree 6, Not recorded/missing |
| I believe air filters reduce infections being spread through the air | 1, Strongly disagree 2, Slightly disagree 3, Not sure 4, Slightly agree 5, Strongly agree 6, Not recorded/missing |
| How would you describe your satisfaction with your care home in terms of…. |  |
| Care home temperature | 1, Very satisfied 2, Satisfied 3, Not sure 4, Dissatisfied 5, Very dissatisfied 6, Not recorded/missing |
| Odours | 1, Very satisfied 2, Satisfied 3, Not sure 4, Dissatisfied 5, Very dissatisfied 6, Not recorded/missing |
| Air quality | 1, Very satisfied 2, Satisfied 3, Not sure 4, Dissatisfied 5, Very dissatisfied 6, Not recorded/missing |
| Would you like to tell us anything else about the above items? | 1, Yes- Free text bow, 2, No |
| The following questions are for staff in intervention care homes ONLY | |
| Since the air filters were installed: |  |
| How would you describe your overall satisfaction with the air filters in your care home? | 1, Very satisfied 2, Satisfied 3, Not sure 4, Dissatisfied 5, Very dissatisfied 6, Not recorded/missing |
| Would you like to tell us anything else about the filters | 1, Yes- Free text bow, 2, No |
| Name of care home | Free text |
| Date completed | dd/mm/yyyy |
